# The TPH1 rs211105 gene polymorphism affects abdominal symptoms and quality of life of diarrhea-predominant irritable bowel syndrome

**DOI:** 10.3164/jcbn.17-76

**Published:** 2018-01-27

**Authors:** Ryo Katsumata, Akiko Shiotani, Takahisa Murao, Manabu Ishii, Minoru Fujita, Hiroshi Matsumoto, Ken Haruma

**Affiliations:** 1 Division of Gastroenterology, Department of Internal Medicine, Kawasaki Medical School, 577 Matsushima, Kurashiki City, Okayama 710-0192, Japan; 2 Department of General Internal Medicine 2, Kawasaki Medical School, 577 Matsushima, Kurashiki City, Okayama 710-0192, Japan

**Keywords:** irritable bowel syndrome, quality of life, serotonin, tryptophan hydroxylase, polymorphism

## Abstract

The gastrointestinal symptoms of irritable bowel syndrome are strongly related to impaired quality of life (QOL), especially in diarrhea-predominant. The gene polymorphisms associated with serotonin, or 5-hydroxytryptamine, alter gastrointestinal symptoms and mental status. We aimed to evaluate the effects of gene polymorphisms on gastrointestinal symptoms, psychological conditions, and QOL, and compare these between patients with diarrhea-predominant irritable bowel syndrome (*n* = 62) and healthy controls (*n* = 64). The gene polymorphisms of 5-HTTLPR, 5-HTTVNTR, TPH1 rs453773, and TPH1 rs211105 were evaluated. Gastrointestinal symptoms, depressive state, and QOL were assessed using the Gastrointestinal Symptom Rating Scale, Self-rating Depression Scale, and Short-Form-36. Gene polymorphisms did not significantly differ in frequency between the two groups. The scores for diarrhea, abdominal pain, and indigestion significantly correlated with the physical component summary score. Only the group of patients with diarrhea-predominant irritable bowel syndrome showed a significant correlation between the TPH1 rs211105 T/T genotype and lower scores for role physical and mental health, and higher scores for indigestion and diarrhea. 5-HTTLPR l/s was associated with lower score of role emotional in the diarrhea-predominant irritable bowel syndrome and higher scores in the controls. The gene polymorphisms of 5-hydroxytryptamine signaling effected gastrointestinal symptoms and QOL, especially of the patients with diarrhea-predominant irritable bowel syndrome.

## Introduction

Irritable bowel syndrome (IBS) is a chronic gastrointestinal (GI) disorder that is characterized by an altered bowel habit and stool forms, in addition to abdominal symptoms such as abdominal pain and bloating, with no detectible organic trigger.^(1,2)^ Although this disease is not thought to be life-threatening, it is associated with impaired quality of life (QOL), especially in diarrhea-predominant irritable bowel syndrome (IBS-D).^(3,4)^ In addition to impairment of QOL, the economic burden of patients with IBS-D was estimated to be approximately 30% greater than that of healthy subjects.^(5)^

Many factors are involved in the pathogenesis of IBS, including serotonin [5-hydroxytryptamine (5-HT)] signaling, low-grade inflammation, visceral hypersensitivity, increased permeability, and micro biota.^(6)^ The genetic variants of IBS, some of which are associated with these factors, have been widely reported.^(7–9)^ As 5-HT signaling strongly contributes to the pathophysiology of IBS by regulating GI motility and perception,^(10)^ the gene polymorphisms associated with 5-HT signaling, such as variants of 5-HT transporter (5-HTT) and tryptophan hydroxylase (TPH), have received remarkable attention.

5-HTT, also known as serotonin transporter (SERT) plays a role in the reuptake of 5-HT in a presynaptic membrane, which leads to the regulation of serotonergic signaling.^(11)^ Several polymorphisms in 5-HTT (SLC6A4) are reported to be associated with the regulation of 5-HTT in the gut. The 5-HTT gene-linked polymorphic region (5-HTTLPR) is composed of 44 base pairs (bp) of insertion/deletion, creating long (l) and short (s) allelic variants. Another well-known polymorphism site is the variable number of tandem repeats (VNTR) located in intron 2, containing 9, 10, or 12 copies of a 17-bp repeat element.^(12)^ Several previous research studies were conducted to evaluate the relationship between polymorphisms of SERT and IBS. Past studies indicated a variation of 5-HTTLPR associated with an IBS subtype.^(13,14)^ Furthermore, gene polymorphisms of SERT were revealed to be related to stress susceptibility.^(15)^

TPH is the rate-limiting enzyme of 5-HT biosynthesis, which has two isoforms.^(16)^ TPH1 is predominantly expressed in peripheral organs, especially enterochromaffin cells in the gut, pineal gland, and spleen, whereas TPH2 is mainly expressed in the enteric nerve and central neurons.^(17)^ Based on the fact that the TPH1 inhibitor LX1031 relieves the symptoms of patients with IBS, TPH function might be deeply involved in IBS pathogenesis.^(18)^ TPH1 gene variants are also associated with mental illnesses such as major depression.^(19)^ Although a report indicated that TPH1 gene polymorphisms at intron 3 rs21105 and TPH1 promoter rs4537731 were related to bloating, diarrhea, and watery stool,^(20)^ little has been known about the relationship between 5-HT signaling-related gene polymorphisms and symptoms of IBS patients. Furthermore, no report has assessed the effect of the gene polymorphisms on abdominal symptoms and mental status simultaneously.

The aim of this research was to evaluate how gene polymorphisms related with 5-HT signaling affect the GI symptoms and QOL of Japanese patients with IBS-D.

## Materials and Methods

This study was a cross-sectional research conducted in Japan. The ethical committee of the Kawasaki Medical School approved the study protocol. Written informed consent was obtained from each research subject before enrollment.

### Subjects

IBS-D patients were diagnosed and classified using the Rome III criteria among outpatient clinic patients in Kawasaki Medical School Hospital between February 2010 and March 2016.^(21)^ Those with other subtypes of IBS were excluded. Previous studies of 5-HT_3_ antagonist suggested that enhanced 5-HT signaling was strongly related to the diarrhea symptoms in patients with IBS.^(22)^ Furthermore, TPH1 gene polymorphisms affected the scores for diarrhea and watery stool,^(20)^ and the effectiveness of the TPH1 inhibitor LX1031 was elucidated in patients with IBS without constipation.^(18)^ Hence, we focused on the relationship between IBS-D and 5-HT-related gene polymorphisms in this study. Treatments for IBS-D were administered to patients on their physicians’ discretion, in accordance with the Japanese guideline for IBS.^(23)^ Healthy volunteers and subjects who had undergone a routine health checkup and had positive fecal occult blood test results without intestinal findings in screening colonoscopy were included as controls. Their medical histories were reviewed to ensure that they had no past disease and no current continuous treatments. The exclusion criteria were overlapping other functional GI disorders (FGIDs) such as functional dyspepsia (FD) and non-erosive reflux disease (NERD) based on self-report symptoms. We asked relatively open-ended questions such as “What are your symptoms?” and “What is the problem?” Patients who reported that they had chronic symptoms related with FGIDs other than IBS-D, including epigastric pain and heartburn, were excluded. Patients with other organic diseases such as peptic ulcer, inflammatory bowel disease, malignant tumor, gallbladder disorder, pancreatitis, or liver disease were also excluded.

### Gene polymorphisms

Peripheral blood sample was drawn to examine gene polymorphisms. All the patients’ genomic DNA was extracted from 200 µl of blood sample in EDTA by using a DNA extraction kit (FAVORGEN, Ping-Tung, Taiwan). Polymerase chain reactions (PCRs), PCR-restriction fragment length polymorphisms (RFLPs), or direct sequencing were performed to identify polymorphisms of TPH1 rs211105 (11:g.18033757T>G) located within intron 3, rs4537731 (11:g.18047335A>G) located within the promoter region at –1066, insertion/deletion polymorphisms in 5-HTTLPR, and VNTR counts in 5-HTT, as previously reported.^(24)^ The specimens for direct sequencing were run on an Applied Biosystems 3130xl Genetic Analyzer (Applied Biosystems, Invitrogen Life Technologies, Carlsbad, CA), conforming with the manufacturer’s recommendations.

### Questionnaire

All the questionnaires were taken before the medical examination in the morning, at the outpatient clinic. Clinical GI symptoms were assessed using the GI Symptom Rating Scale (GSRS), which consists of 15 questions rated on a scale of 1 to 7. The questions can be used to evaluate the following five major GI symptoms: abdominal pain, reflux syndrome, diarrhea syndrome, indigestion syndrome, and constipation syndrome. Higher scores indicated more uncomfortable symptoms.^(25)^ QOL was assessed using Short-Form 36 (SF-36), which includes eight health domains as follows: (1) physical functioning; (2) role limitations by physical problems; (3) bodily pain; (4) general health perceptions; (5) vitality (energy/fatigue); (6) social functioning; (7) role limitations due to emotional problems; and (8) mental health/emotional well-being perception. The test consists of 36 questions, with scores ranging from 0 to 100; higher scores indicate better health. The physical component summary (PCS) and mental component summary (MCS) were calculated using eight domains. The raw scores for the eight categories were linearly transformed with standard scoring algorithms yielding scores that were then further adjusted using a Japanese norm-based scoring system (NBS) to generate normalized scores with a mean (±SD) of 50 ± 10.^(26)^ Depressive status was assessed using the Self-rating Depression Scale (SDS).^(27)^ The scale is composed of 50 standard scoring algorithms yielding scores, and depression was defined as a total score of >50.

### Statistical analysis

Continuous data are expressed as means; and categorical data, as counts and percentages. Normal distribution and homoscedasticity were tested using the Shapiro-Wilk and Levene tests. Two-group comparison was performed with the chi-square analysis for categorical variables, and the unpaired *t* test or Mann-Whitney *U* test for continuous data. Correlation was determined using the Spearman test. All two-sided *p* values of <0.05 were considered significant. All statistical calculations were performed using SPSS ver. 20 for Windows (SPSS Inc, Chicago, IL).

## Results

The study groups consisted of 62 patients with IBS-D (male-to-female ratio, 40:22) and 64 healthy controls (42:22). Demographics, GSRS scores, SF-36 scores, and genotype frequencies are shown in Table 1. Unsurprisingly, the scores for GI symptoms and SDS were higher and the QOL scores were lower in the IBS-D group than in the control group. No significant differences in the frequencies of the genotypes were found between the two groups.

### Correlation between GSRS scores and SF-36 NBS in the IBS-D group

**Fig. 1:**
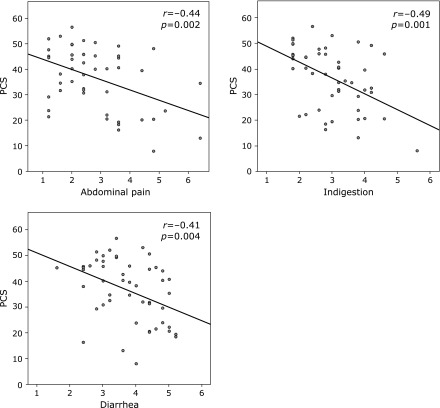
Correlation between GSRS scores and physical component summary (PCS) scores in IBS-D patients. PCS score was negatively correlated with abdominal pain, indigestion, and diarrhea scores. GSRS, gastrointestinal symptom rating scale; SF-36, Short-Form-36; IBS-D, diarrhea-predominant irritable bowel syndrome. Correlation coefficient was described as *r* value. *p* and *r* values were calculated by Spearman’s test.

Excluding constipation, most domains of the SF-36 NBS negatively correlated with the GSRS scores (reflux, abdominal pain, indigestion, and diarrhea). Not only the individual domains but also the PCS scores correlated with abdominal symptoms such as abdominal pain, indigestion, and diarrhea (Fig. 1).

### Association of the 5-HTT genotypes

The mean role emotional NBS score was significantly lower (45.1 vs 52.1, *p* = 0.02) in the controls with 5-HTTVNTR s/s than in the controls with l/s, although it was significantly higher (45.0 vs 35.3, *p* = 0.04) in the IBS-D patients with s/s than in those with l/s (Table 2). In the IBS-D group, the median GSRS score for reflux was significantly lower (1.5 vs 2.3, *p* = 0.02) in the patients with s/s than in the patients with l/s. The 5-HTTVNTR genotypes were significantly associated with the SF-36 scores in the control group but not in the IBS-D group. The mean NBS scores for physical functioning, role physical, body pain, vitality, role emotional, and physical component summary were significantly lower in the controls with 5-HTTVNTR 12/12 than in the controls with 12/10. However, no significant association was found between the 5-HTTVNTR genotypes and the GSRS scores. Depressive scores tended to be higher in the controls with 5-HTTVNTR 12/12 than in the controls with 12/10, but the difference was not significant.

### Association of the TPH1 genotypes

The frequencies of the TPH1 rs4537731 minor G allele and rs211105 minor G allele in the IBS group were respectively 33.8% and 22.5%, and 42.1% and 28.1% in the control group (Table 3). The TPH1 rs211105 T homo-genotype was significantly associated with not only the indigestion and diarrhea GSRS scores but also the role physical and mental health SF-36 scores only in the IBS-D group. The median scores for indigestion (2.8 vs 1.9, *p* = 0.01) and diarrhea (5.3 vs 3.2, *p* = 0.02) were significantly higher and the mean NBS scores for role physical (36 vs 47.2, *p* = 0.01) and mental health (38.7 vs 47, *p* = 0.01) were significantly lower in patients with IBS-D with the T/T genotype than in those with the T/G genotype. However, no association between TPH1 rs4537731 SNP and the scores for GI symptoms and QOL was found in both groups. SDS scores tended to be higher in patients with IBS-D with the T/T genotype than in those with the T/G genotype, but the difference was not significant between the two groups.

## Discussion

We revealed that the frequencies of the SERT and TPH1 gene polymorphisms were not significantly different between patients with IBS-D and healthy controls, and that the TPH1 rs211105 T homo genotype was significantly associated with severe QOL and IBS symptoms in our patients with IBS-D. The frequencies of the SERT and TPH1 polymorphisms in patients with IBS in comparison with those in healthy controls are still controversial, although several studies were reported to date. The prevalence of TPH1 gene polymorphisms, including rs211105 and rs453771, did not differ between patients with IBS and healthy subjects.^(20,28)^ Furthermore, in a meta-analysis, no significant difference was detected in the prevalence of 5-HTTLPR variation between patients with IBS and healthy controls.^(29)^ Considering the results of past studies and this study, the polymorphisms of SERT and TPH1 in patients with IBS-D seem not crucial to the onset of IBS-D but is a possible factor that modifies GI symptoms and mental status in Japanese patients.

Although the frequencies of TPH1 rs211105 and rs453771 gene polymorphisms were not significantly different between patients with IBS and controls, the mean diarrhea and indigestion scores were significantly higher in patients with IBS-D with the TPH1 rs211105 T/T genotype than in those carrying the minor G allele. Besides, mean scores of role physical and mental health were significantly lower in the IBS-D patients with the T/T genotype than in those with the T/G genotype. The impact of the TPH1 rs211105 variant has not been fully elucidated so far. A previous study reported that Caucasian female patients with IBS and the TPH1 rs211105 T/G genotype showed more severe abdominal symptoms than patients with the T/T genotype.^(20)^ Conversely, even among Caucasian patients with IBS, Jun *et al.*^(30)^ detected an association between the TPH1 rs211105 T/T genotype and impairment of IBS-related cognition, which affects IBS symptoms. Furthermore, we reported that the TPH1 rs211105 T/T genotype in Japanese patients with IBS was associated to the efficacy of ramosetron, a 5-HT3 antagonist.^(23)^ This result and our findings implied that upregulated 5-HT signaling is evoked in TPH1 rs211105 T/T subjects. Indeed, increased 5-HT concentration in intestines induces drastic changes in motility and perception, which result in GI symptoms.^(2)^ 5-HT shortens the transit time in the small bowel and enhances fluid secretion in the human jejunum.^(31,32)^ These functions can cause diarrhea. Short transit time and increased intestinal fluid secretion might lead to increased flatus and abdominal distention, respectively, which cause higher indigestion scores. Thus, the TPH1 rs211105 T/T genotype in Japanese patients might cause enhanced 5-HT signaling that leads to abdominal symptoms.

In our study, the 5-HTTLPR s/s genotype indicated less severe reflux symptom and higher QOL score. Several researchers investigated the relationship between 5-HTTLPR polymorphisms and abdominal symptoms. Colucci *et al.*^(33)^ reported that Caucasian patients with IBS and 5-HTTLPR s/s and l/s had greater abdominal pain than patients with 5-HTTLPR l/l. Unlikely abdominal symptoms in our patients were not different between s/s and L/s. The inconsistency of race and subtype might have influenced this discrepancy. Regarding reflux symptoms, a few research studies indicated a relationship between 5-HTTLPR polymorphism and gastroesophageal reflux disease (GERD) symptoms. In an animal experiment, the relative expression levels of SERT in the esophageal mucosa were higher in subjects with reflux esophagitis (RE) and NERD.^(34)^ An elevated SERT expression level induces a reduction in 5-HT concentration, thereby inhibiting esophageal motility and acid clearance ability. The 5-HTTLPR l/l genotype has greater transcriptional activity, which results in a higher 5-HTT expression level in a synaptic cleft, than the 5-HTTLPR s/s genotype.^(35)^ Therefore, the 5-HTTLPR l/s genotype associated with higher SERT expression level and lower 5-HT level at the synaptic cleft might have exacerbated the GERD symptoms, followed by QOL impairment, in our subjects. However, further investigations about the relationship between SERT polymorphisms and GERD symptoms are still needed to disclose the mechanisms.

The correlation between abdominal symptoms and QOL scores was confirmed in our patients with IBS-D. Patients with IBS have been reported to have lower QOL scores than those with other chronic diseases.^(36)^ In patients with IBS, as symptoms worsen, QOL scores tend to decrease.^(37)^ In addition, a previous meta-analysis indicated that the depression and anxiety levels in patients with IBS were higher than those in healthy controls.^(38)^ These strong relevance between digestive symptoms and mental status is a part of the brain-gut axis, which is a well-known bidirectional effect between the gastro-intestine and the brain.^(39)^ As with past studies, we confirmed that the severity of abdominal symptoms in Japanese patients with IBS-D was associated with impaired QOL.

When it comes to the treatment for IBS-D patients, not only GI symptoms but also mental status should be considered as therapeutic targets. In addition to treatment aimed at GI symptoms, other approaches that target mental dysfunction would be feasible options. Indeed, cognitive behavioral therapy was elucidated to be effective for patients with IBS.^(40)^ 5-HT signaling is the reasonable target associated with GI symptoms and mental manifestation. Inhibition of TPH1 improved the symptoms in patients with IBS to a certain extent.^(18)^ In this study, we detected a specific cluster that has enhanced 5-HT signaling and better therapeutic effect of an TPH1 inhibitor.

This study has several limitations. First, possible selection bias especially related to the relatively small number of subjects might have affected our results. The prevalences of 5-HTTLPR and TPH1 polymorphisms differ among various populations. A large-scale multi-country study is required to eliminate the racial influence. Another limitation is the flexible treatment for patients with IBS-D. Moreover, the data on abdominal symptoms were limited. We evaluated symptoms by using only a questionnaire. Other objective data such as stool frequency and shape of stool would be effective for further investigation. We did not establish a protocol for a standard treatment instead of the physician’s choice. However, it was ethically inadequate to stabilize therapeutic options. Lastly, we did not assess the local environment, including TPH1 expression and 5-HT concentration, as described in other studies.^(24,41)^ Even though these methods would be effective to reveal the connecting mechanism between genetic variations and abdominal symptoms, evaluation of gene polymorphisms is feasible and sufficient to predict the severity of abdominal symptoms and QOL.

In conclusion, we elucidated that the TPH1 rs211105 variant affected abdominal symptoms and QOL. Our findings in this research shed light on the pathogenesis of IBS-D, and the TPH1 rs211105 variant might be a biomarker of enhanced 5-HT signaling that leads to severe GI symptoms and worsening of QOL in Japanese patients with IBS-D.

## Author Contributions

Ryo Katsumata performed the experiments on gene polymorphisms, analyzed the data, and wrote the manuscript. Akiko Shiotani conceptualized the study and revised the manuscript. Hiroshi Matsumoto contributed to discussion of the results of the study. Akiko Shiotani, Hiroshi Matsumoto, Takahisa Murao, Manabu Ishii, Minoru Fujita, and Ken Haruma collected the clinical data and blood samples. All the authors read and approved the final version of this paper.

## Figures and Tables

**Table 1:** Demographics data, scores of GSRS, SF-36, and frequency of gene polymorphisms in IBS-D patients and healthy controls

	Controls (*n* = 64)	IBS-D (*n* = 62)	*p*
Age mean (SD)	39.7 (14.5)	43.3 (17.4)	0.53^a^
Gender male (%)	42 (65.6)	40 (64.5)	0.76^b^
GSRS median (IQL)			
Reflux	1 (1–1.5)	2 (1–3)	**<0.01**^c^
Abdominal pain	1 (1–1.7)	3 (1.7–3.9)	**<0.01**^c^
Indigestion	1.3 (1–1.8)	2.4 (1.5–3.5)	**<0.01**^c^
Diarrhea	1 (1–1.7)	5 (3.3–6)	**<0.01**^c^
Constipation	1.3 (1–2)	2.3 (1.7–3)	**<0.01**^c^
SF-36 NBS mean (SD)			
PF	55.0 (5.0)	52.3 (7.5)	**0.02**^a^
RP	48.9 (12.6)	38.7 (16.7)	**<0.01**^a^
BP	53.3 (9.8)	41.2 (11.6)	**<0.01**^a^
GH	52.0 (10.9)	40.8 (11.6)	**<0.01**^a^
VT	48.1 (10.3)	42.3 (10.8)	**<0.01**^a^
SF	49.2 (12.8)	40.3 (13.3)	**<0.01**^a^
RE	47.4 (13.0)	41.7 (14.9)	**0.03**^a^
MH	49.5 (11.2)	40.7 (10.7)	**<0.01**^a^
PCS	51.9 (9.2)	44.8 (13.6)	**<0.01**^a^
MCS	49.4 (4.5)	47.7 (3.6)	**0.04**^a^
SDS mean (SD)	36.8 (7.8)	44.0 (8.9)	**<0.01**^a^
Genotype			
5-HTTLPR del(s)/ins(l) s/s, l/s, l/l	42, 19, 3	41, 21, 0	0.15^b^
5-HTT intron2 VNTR 12/12, 10/12rep	52, 12	50, 12	0.81^b^
TPH1 rs4537731 -6526 A>G A/A, A/G, G/G	39, 27, 0	41, 20, 1	0.38^b^
TPH1 rs211105 intron3 T>G T/T, T/G, G/G	46, 18, 0	48, 13, 1	0.41^b^

GSRS, gastrointestinal symptom rating scale; SF-36, Short-Form-36; IBS-D diarrhea-predominant irritable bowel syndrome; NBS, norm-based scoring; PF, physical functioning; RP, role physical; BP, bodily pain; GH, general health; VT, vitality; SF, social functioning; RE, role emotional; MH, mental health; PCS, physical component summary; MCS, mental component summary; SDS, Self-rating Depression Scale. *P* values calculated using a, unpaired *t* test; b, chi-squared test; c, Mann-Whitney *U* test.

**Table 2:** Association of polymorphisms of *5-HTT* with GSRS and SF-36 scores

5-HTTLPR	Controls		*p*	IBS-D		*p*
	s/s (*n* = 42)	l/s (*n* = 22)		s/s (*n* = 41)	l/s (*n* = 21)	
Age mean (SD)	40.7 (15.2)	37.8 (11.4)	0.64^a^	41.8 (19.3)	47.7 (12.9)	0.96^a^
Gender male (%)	31 (73.1)	11 (50)	0.13^b^	24 (58.5)	16 (76.1)	0.26^b^
GSRS median (IQL)						
Reflux	1 (1–1.8)	1 (1–1.5)	0.35^c^	1.5 (1–2)	2.3 (1–4.1)	**0.02**^c^
Abdominal pain	1 (1–1.3)	1.7 (1–1.8)	0.07^c^	2 (1.7–3)	2 (1.6–3.5)	0.58^c^
Indigestion	1.3 (1–1.9)	1.4 (1–1.8)	0.62^c^	2.3 (1.3–3.5)	2.7 (1.5–3.1)	0.85^c^
Diarrhea	1 (1–1.3)	1.5 (1–2.1)	0.18^c^	5 (3.4–6)	4.7 (3.3–5.6)	0.54^c^
Constipation	1.3 (1–1.8)	1.7 (1–2.1)	0.12^c^	2.3 (1.7–3.6)	2.5 (1.3–3.3)	0.57^c^
SF-36 NBS mean (SD)						
PF	54.5 (5.1)	56.1 (4.7)	0.28^a^	52.8 (7.8)	52.4 (7.3)	0.93^a^
RP	47.3 (14.5)	52.3 (6.8)	0.09^a^	39.4 (15.7)	37.5 (18.9)	0.68^a^
BP	51.9 (10.6)	56.3 (7.3)	0.11^a^	41.3 (11.1)	41.1 (12.8)	0.95^a^
GH	50.9 (11.7)	54.2 (9)	0.31^a^	41.6 (10.9)	39.4 (12.2)	0.49^a^
VT	46.8 (9.9)	50.8 (10.8)	0.17^a^	43.5 (10.1)	40.1 (11.8)	0.25^a^
SF	49.3 (13.1)	49.1 (12.4)	0.95^a^	42 (12.2)	37 (14.8)	0.17^a^
RE	45.1 (14.3)	52.1 (8.4)	**0.02**^a^	45 (11.1)	35.3 (18.9)	**0.04**^a^
MH	47.5 (11.6)	53.7 (9.5)	0.06^a^	41.2 (10.2)	39.8 (11.8)	0.64^a^
PCS	50.6 (11.1)	54.4 (3.6)	0.07^a^	46.5 (11.8)	49.7 (10.3)	0.34^a^
MCS	49.1 (5.9)	46.6 (4.7)	0.12^a^	50.8 (5.1)	48.1 (5.4)	0.06^a^
SDS mean (SD)	37.4 (8.0)	35.5 (7.6)	0.38^a^	42.3 (7.8)	47.1 (10.2)	0.58^a^

5-HTTVNTR	Controls		*p*	IBS-D		*p*
	12/12 (*n* = 50)	10/12 (*n* = 12)		12/12 (*n* = 50)	10/12 (*n* = 12)	
Age mean (SD)	39.7 (14.8)	40.1(9.9)	0.81^a^	43 (17.5)	47.2 (17.7)	0.41^a^
Gender male (%)	33 (63.4)	9 (75)	0.71^b^	31 (62)	9 (75)	0.51^b^
GSRS median (IQL)						
Reflux	1 (1–1.6)	1 (1–1.3)	0.39^c^	1.8 (1–2.6)	2 (2–3.9)	0.15^c^
Abdominal pain	1 (1–1.7)	1.7 (1–2.3)	0.09^c^	2 (1.6–3)	2.2 (2–3.9)	0.23^c^
Indigestion	1 (1–2.1)	1.5 (1–1.5)	0.96^c^	2.3 (1.4–3.5)	3.3 (1.8–3.9)	0.27^c^
Diarrhea	1 (1–1)	1.3 (1–3)	0.45^c^	4.8 (3.3–6.1)	5.5 (4.4–5.7)	0.63^c^
Constipation	1 (1–1.3)	1.3 (1–1.7)	0.61^c^	2.3 (1.7–3)	2.7 (1.3–4.3)	0.76^c^
SF-36 NBS mean (SD)						
PF	54.6 (5.3)	57.5 (1.8)	**0.01**^a^	51.8 (8)	54.4 (5.2)	0.31^a^
RP	47.8 (13.5)	54.7 (3.5)	**0.01**^a^	40.5 (16)	31.1 (18.6)	0.09^a^
BP	52 (10.2)	59.8 (3.1)	**<0.01**^a^	41.8 (11.4)	38.7 (12.2)	0.43^a^
GH	51.7 (11.4)	54 (8.4)	0.59^a^	41 (11.1)	40.4 (12.7)	0.88^a^
VT	46.6 (10.4)	55.4 (6.1)	**0.01**^a^	42.2 (10.5)	43 (12.3)	0.83^a^
SF	48.2 (13.3)	54.2 (8.8)	0.20^a^	40.9 (12.5)	38 (16.7)	0.52^a^
RE	45.6 (13.5)	56.6 (0.02)	**<0.01**^a^	42.6 (14.2)	38 (17.7)	0.36^a^
MH	48.5 (11.7)	54.7 (7.1)	0.12^a^	41.6 (10)	36.8 (13)	0.17^a^
PCS	51.6 (7.8)	56.6 (2.6)	**<0.01**^a^	47.5 (12.1)	46.9 (6.7)	0.85^a^
MCS	48.6 (5.8)	46.8 (4.3)	0.35^a^	49.8 (5.3)	50.1 (5.5)	0.88^a^
SDS mean (SD)	37.5 (8.2)	33 (4.1)	0.11^a^	43.2 (8.3)	46.7 (10.7)	0.23^a^

GSRS, gastrointestinal symptom rating scale; SF-36, Short-Form-36; NBS, norm-based scoring; PF, physical functioning; RP, role physical; BP, bodily pain; GH, general health; VT, vitality; SF, social functioning; RE, role emotional; MH, mental health; IBS-D, diarrhea-predominant irritable bowel syndrome; PCS, physical component summary; MCS, mental component summary; SDS, Self-rating Depression Scale. *P* values calculated using the a, unpaired *t* test; b, chi-squared test; c, Mann-Whitney *U* test.

**Table 3:** Association of polymorphisms of TPH1 rs211105, GI symptoms, and QOL scores

TPH1 rs211105	Controls		*p*	IBS-D		*p*
	T/T (*n* = 46)	T/G (*n* = 18)		T/T (*n* = 48)	T/G (*n* = 14)	
Age mean (SD)	40.9 (13.6)	37.5 (15.3)	0.89^a^	41.7 (17.8)	51 (14.6)	0.64^a^
Gender male (%)	30 (65.2)	12 (66.7)	0.92^b^	33 (68.7)	7 (50)	0.21^b^
GSRS median (IQL)						
Reflux	1 (1–1.5)	1.5 (1–1.9)	0.69^c^	2 (1–3)	2 (1–2.5)	0.76^c^
Abdominal pain	1 (1–1.7)	1 (1–1.7)	0.61^c^	2 (1.4–3.5)	2.2 (1.9–3)	0.84^c^
Indigestion	1.3 (1–1.8)	1.5 (1.1–2.1)	0.24^c^	2.8 (1.5–3.8)	1.9 (1–2.4)	**0.01**^c^
Diarrhea	1 (1–2)	1.2 (1–1.7)	0.92^c^	5.3 (3.8–6)	3.2 (2.3–5.7)	**0.02**^c^
Constipation	1.3 (1–2)	1.3 (1–1.7)	0.54^c^	2.7 (2–3.3)	1.8 (1.3–3)	0.12^c^
SF-36 NBS mean (SD)						
PF	55 (4.9)	55.1 (5.5)	0.95^a^	52.6 (7.1)	51.4 (9.1)	0.58^a^
RP	48.3 (12.5)	50.4 (13.3)	0.56^a^	36 (17.3)	47.2 (11.5)	**0.01**^a^
BP	52.1 (10.6)	56.2 (6.9)	0.09^a^	40.7 (11.8)	42.8 (11.2)	0.56^a^
GH	52.5 (10.9)	56.2 (6.9)	0.67^a^	40.2 (11.5)	42.8 (10.8)	0.45^a^
VT	48.3 (11.1)	47.6 (8.4)	0.81^a^	40.8 (10.8)	47.2 (9.3)	0.05^a^
SF	48 (14)	52.2 (8.8)	0.19^a^	39.3 (14.4)	43.5 (8.3)	0.19^a^
RE	47.1 (13)	48.1 (13.4)	0.80^a^	41 (15.2)	43.8 (14.1)	0.54^a^
MH	49 (11.7)	50.8 (10.2)	0.59^a^	38.7 (10.7)	47 (8.4)	**0.01**^a^
PCS	51.8 (7.5)	54.2 (7.1)	0.34^a^	45.7 (12.4)	49.1 (10.3)	0.33^a^
MCS	49.1 (5.7)	48.9 (5.4)	0.64^a^	48.5 (3.6)	49.3 (5.4)	0.58^a^
SDS mean (SD)	36.7 (8.3)	36.8 (7.1)	0.97^a^	44.8 (8.5)	41 (9.9)	0.19^a^

GSRS, gastrointestinal symptom rating scale; SF-36, Short-Form-36; NBS, norm-based scoring; PF, physical functioning; RP, role physical; BP, bodily pain; GH, general health; VT, vitality; SF, social functioning; RE, role emotional; MH, mental health; IBS-D, diarrhea-predominant irritable bowel syndrome; PCS, physical component summary; MCS, mental component summary; SDS, Self-rating Depression Scale. *P* values calculated using the a, unpaired *t* test; b, chi-squared test; c, Mann-Whitney *U* test.

## Abbreviations

GERD: gastroesophageal reflux disease
GSRS: gastrointestinal symptom rating scale
5-HT: 5-hydroxytryptamine
5-HTT: 5-hydroxytryptamin transporter
5-HTTLPR: 5-hydroxytryptamin transporter gene-linked polymorphic region
IBS: irritable bowel syndrome
IBS-D: diarrhea-predominant irritable bowel syndrome
NBS: norm-based scoring system
MCS: mental component summary
PCS: physical component summary
QOL: quality of life
SERT: serotonin transporter
TPH: tryptophan hydroxylase
VNTR: variable number of tandem repeats
